# A Randomized Clinical Trial of *Schinus terebinthifolius* Mouthwash to Treat Biofilm-Induced Gingivitis

**DOI:** 10.1155/2013/873907

**Published:** 2013-06-17

**Authors:** Irlan de Almeida Freires, Livia Araújo Alves, Gabriela Lacet Silva Ferreira, Vanessa de Carvalho Jovito, Ricardo Dias de Castro, Alessandro Leite Cavalcanti

**Affiliations:** ^1^Department of Physiological Sciences, Piracicaba Dental School, State University of Campinas, 13.417-095 Piracicaba, SP, Brazil; ^2^Department of Oral Diagnosis, Piracicaba Dental School, State University of Campinas, 13.417-095 Piracicaba, SP, Brazil; ^3^School of Dentistry, Federal University of Paraiba, 58.035-260 Joao Pessoa, PB, Brazil; ^4^Department of Clinics and Social Dentistry, School of Dentistry, Federal University of Paraiba, 58.038-040 Joao Pessoa, PB, Brazil; ^5^Department of Dentistry, School of Dentistry, State University of Paraiba, 58.038-300 Campina Grande, PB, Brazil

## Abstract

*Objectives*. This study aimed to investigate the efficacy of a *Schinus terebinthifolius* (ST) mouthwash in reducing gingival inflammation levels (GI) and biofilm accumulation (BA) in children with gingivitis. *Methods*. This was a randomized, controlled, triple blind, and phase II clinical trial, with children aged 9–13 years (*n* = 27) presenting with biofilm-induced gingivitis. The sample was randomized into experimental (0.3125% ST, *n* = 14) and control (0.12% chlorhexidine/CHX, *n* = 13) groups. Products were masked as regards color, flavor and aroma. Intervention protocol consisted in supervised rinsing of 10 mL/day for 01 minute for 10 days. Gingival bleeding and simplified oral hygiene indexes were used to assess the efficacy variables, measured at baseline and after intervention by calibrated examiners. Data were statistically treated with paired *t*-test, unpaired *t*-test, and Wilcoxon and Mann-Whitney tests (**α** = .05). *Results*. It was found that both ST and CHX were able to significantly reduce GI levels after 10 days (*P* < 0.001) and there was no significant difference between them (*P* > 0.05). CHX was the only product able to significantly reduce BA after 10 days when compared to baseline (*P* < 0.05). *Conclusion*. ST mouthwash showed significant anti-inflammatory activity (equivalent to CHX), but it was not able to reduce biofilm accumulation.

## 1. Introduction

Periodontal disease refers to a spectrum of clinical manifestations that affect tooth protection tissues, classified as gingivitis, or may also damage support structures, summarily termed as periodontitis [1]. It has been long known that periodontal impairment onset is primarily attributed to biofilm accumulation [2, 3] or can also be related to systemic diseases such as diabetes and leukemia, medications, and malnutrition [1].

Biofilm-induced gingivitis has been widely spread among children and adolescents worldwide [4–6], with especial attention to individuals about 12 years old whose disease prevalence has reached over 77% [7].

Accordingly, children have been considered a group favorable to develop gingivitis in the population [5] due to lack of maturity in understanding self-care importance, anatomophysiological peculiarities, and erupting teeth, among other factors. In this respect, early diagnosis and treatment of gingival inflammation are essential to prevent the establishment of clinical periodontitis [8].

Overall, periodontal disease prevention has been known to require constant dental plaque removal, either by mechanical (toothbrush) or chemical means—mouthwashes or other topical methods [9].

The foremost chemical agents currently available are chlorhexidine, triclosan, cetylpyridinium chloride, and natural products [10, 11]. In this respect, natural products have been proposed in an attempt to minimize undesirable effects caused by synthetic agents such as tooth staining, imbalance of the resident microbiota, and altered taste [12], besides representing a new antimicrobial possibility that might be used to face a situation of microbial resistance.

Thus, medicinal plants have been investigated as a natural resource to treat microbial infections and also subsidize the development of new drugs with specific therapeutic properties [13].

 Among several plant species presenting biological activities, *Schinus terebinthifolius*, popularly known as Brazilian pepper tree, calls attention. *S. terebinthifolius* is native to South America and belongs to the plant kingdom, division Tracheophyta, class Agnoliopsida, order Sapindales, and family Anacardiaceae [14]. This species which has been proven to present antimicrobial [15], anti-inflammatory [16], and antiulcerogenic [17] effects, is used as antiseptic and in the treatment of stomatitis. Its leaves have been commonly used worldwide in the management of venereal diseases, womb inflammation, urinary tract infections, skin wounds, diarrhea, and gastroduodenal ulcer [18].

In this perspective, the present controlled trial investigated the clinical efficacy of an experimental mouthwash containing *S. terebinthifolius* (Brazilian pepper tree) in reducing biofilm-induced gingivitis levels in children aged 9–13 years.

## 2. Material and Methods

### 2.1. Ethical Issues and Trial Registration

 Prior to execution, this study was approved by a Brazilian Ethics Committee at Lauro Wanderley University Hospital, Joao Pessoa, PB, Brazil (Protocol no. 159/10). A letter containing the study description was delivered to the children's parents asking for their authorization by signing the informed-consent form (only whether participation was consented). This research has followed the guidelines of the 196/96 Resolution of the Brazilian National Health Council, which encompasses Helsinki's Declaration.

 This trial was registered in 2009 on the Clinicaltrials.gov (US National Institute of Health Registry) under protocol NCT 01197105.

### 2.2. Study Design

This was a phase II clinical investigation, in which efficacy and safety of the experimental product were determined upon a relatively small number of diseased individuals [19]. The CONSORT guidelines were followed to design this investigation.

#### 2.2.1. Randomization

Sample allocation into the treatment groups (control and experimental) was based on computer-generated sequences. The allocation concealment was performed by a single researcher who did not examine the subjects. Data were reserved during study design and execution under a single researcher's responsibility.

#### 2.2.2. Control

It consisted of individuals who used 0.12% chlorhexidine digluconate mouthwash (gold standard), manipulated in a compounding pharmacy in accordance with the authors' requirements.

#### 2.2.3. Blinding

Experimental and control products were kept in similar containers, and the liquid content presented the same color (reddish), taste, and smell (strawberry). The justification for this procedure lies in the potential for bias that occurs when all subjects involved in the trial know which treatments they are receiving [20]. Accordingly, neither children nor examiners knew in which group the subjects were enrolled, and the statistician also did not know to which arm belonged the data under analysis, so characterizing the study as triple blind.

#### 2.2.4. Intra- and Interexaminer Agreement

Two examiners were previously calibrated as regards gingival inflammation and biofilm accumulation evaluations [21]. *Kappa* values obtained are disposed in Table 1.

### 2.3. Study Participants

Eligibility criteria were used in order to delimitate and homogenize the sample as follows.

#### 2.3.1. Inclusion Criteria

Children aged 9–13 years old; presence of at least 15 teeth; biofilm-induced gingivitis; informed consent signed by the child's legal guardian.

#### 2.3.2. Exclusion Criteria

Presence of systemic alteration(s) (screened from a rapid questionnaire sent to parents) that might interfere with the periodontal disease course, according to the literature; wearing fixed or removable orthodontic appliances; having used antiseptic mouthwashes in the three months prior to the study; undergoing treatment with antimicrobial or anti-inflammatory drugs.

**Figure 1:**
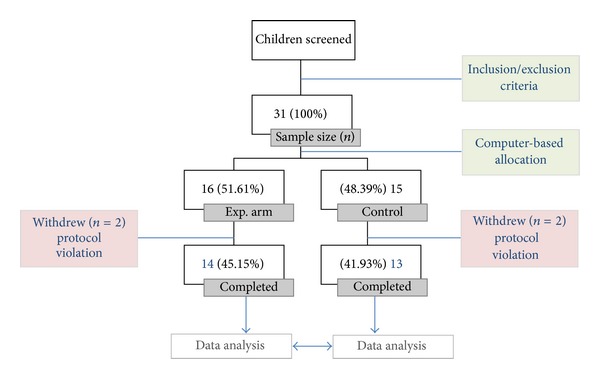
Sample flowchart expressed in absolute and percentage (%) number of subjects.

Sample size estimation was conducted according to the Fleming's single-stage procedure [25]. Response rate has been found to be ~30%. Defining *p*_*o*_ as the proportion of response below which treatment does not warrant further investigation and *p*_*n*_ as the proportion of responses beyond which further trials should be carried out, it was set *p*_*o*_ = 0.3 and *p*_*n*_ = 0.7. Accordingly, a sample size of 13 subjects in each group would provide 92% power (1-*β*) to detect any clinically relevant treatment difference of 30% in relation to baseline at a two-tailed significance level (*α*) of 0.05. Considering a dropout of sample of 20% (~5 subjects), the final sample size consisted of 31 individuals.  Figure 1 expresses the sample flowchart since children's screening until data analysis.

 All individuals included underwent hygiene standardization by receiving an oral hygiene kit containing 30 g of 1400 ppmF dentifrice and toothbrush with soft bristles and small head. No brushing orientation was performed within the study period. Parents and children were advised to keep children's oral care routine as to mitigate the *Hawthorne effect* [26].

### 2.4. Experimental Product

 The mouthwash formulation contained the stem bark tincture of* Schinus terebinthifolius* at 0.3125% (Table 2), which was the lowest *in vitro* concentration capable of inhibiting bacterial growth [27]. The product met all specifications required in relation to its quality control according to analyses previously performed.

 The pH of the experimental and control mouthwashes was set at 7.0, and these were kept in milky white containers at room temperature.

 Phytochemical profile of *S. terebinthifolius* indicated triterpenes [28], flavonoids [28, 29], steroids, saponins [28], and tannins [29]. Due to these phytoconstituents, the species was expected to present antimicrobial and anti-inflammatory properties.

### 2.5. Intervention Protocol

The children were subjected to a 01-minute supervised rinse of 10 mL daily for 10 consecutive days at the same hour (30 minutes before afternoon snack). This intervention was controlled by a single researcher, who did not perform the examinations.

 In the end of the intervention period, children were instructed with regards to brushing techniques and oral care importance.

### 2.6. Efficacy Variables

Clinical examinations were performed using a dental mirror (#5) and WHO periodontal probes adequately sterilized. Data were registered in a clinical record by two trained annotators.  Table 3 brings the efficacy variables evaluated in the study and supplementary information. 

The SOHI was considered to assess the oral hygiene status of the study participants and to keep an account of plaque being the main etiological factor for the onset of periodontal disease. The GBI was considered as an indicator of the inflammatory condition [30].

Examiners did not supervise the rinses and had contact with the subjects only on the examination days.

### 2.7. Adverse Effects

Any adverse effect reported or identified (e.g., tooth staining, mucosal desquamation, etc.) would be duly registered in the clinical record, contemplating:

- intensity (middle, moderate, and severe);
- nature (collateral effect, toxic effect, and meta-reactions);
- onset and duration;
- exact period in which the effect was observed within the 10-day timeline.

### 2.8. Statistical Analysis

Type I error (*α*) was set at 0.05, and type II (*β*) error was established as ≤0.2. In order to verify if gingival bleeding values obeyed to a Gaussian distribution curve, the Kolmogorov-Smirnov with Dallal-Wilkinson test was performed. Then, according to intra- or intergroup comparisons at baseline and after treatment, *paired t* and *unpaired t-*tests were applied. Biofilm accumulation was categorized; therefore, Wilcoxon and Mann-Whitney tests were employed, respectively, to intra- and intergroup comparisons at baseline and after the 10-day treatment. In addition to inferential analyses, descriptive statistics was also applied when necessary. Analyses were performed on GraphPad Prism 5.0 and BioEstat 5.0 software.

## 3. Results

A number of 31 subjects were included in the study, of which 87.09% (*n* = 27) completed the trial (Figure 1).

In the total, the sample was composed of 59.25% females and 40.75% males. Clinical profile is disposed in Table 4 according to treatment arm.

### 3.1. Treatment Outcomes

#### 3.1.1. Gingival Inflammation

**Figure 2:**
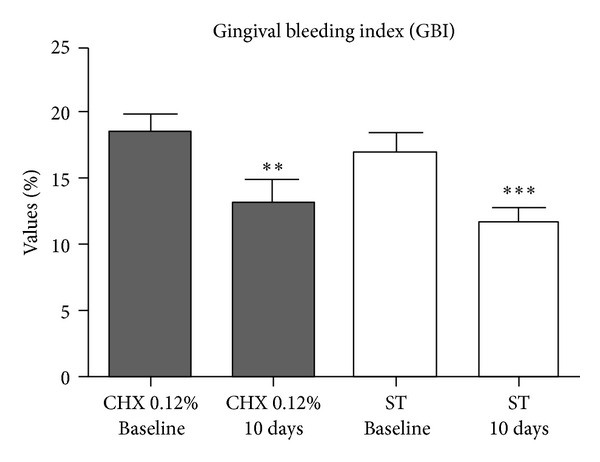
Gingival bleeding index (GBI) values for chlorhexidine 0.12% and *S. terebinthifolius* groups at baseline and after 10-day treatment (**P  value = 0.0027; ***P  value < 0.0001), *paired t-test*.

 As seen in Figure 2 and Table 5, data revealed no statistically significant difference between groups at baseline as regards gingival inflammation (*P* > 0.05). After 10 days, both the control and the experimental groups were able to statistically reduce gingival inflammation levels (*P* < 0.05), and intergroup comparisons showed no difference between 0.12% chlorhexidine and *S. terebinthifolius* efficacy (*P* > 0.05). Means and standard deviations are expressed in Table 5.

#### 3.1.2. Biofilm Accumulation

**Figure 3:**
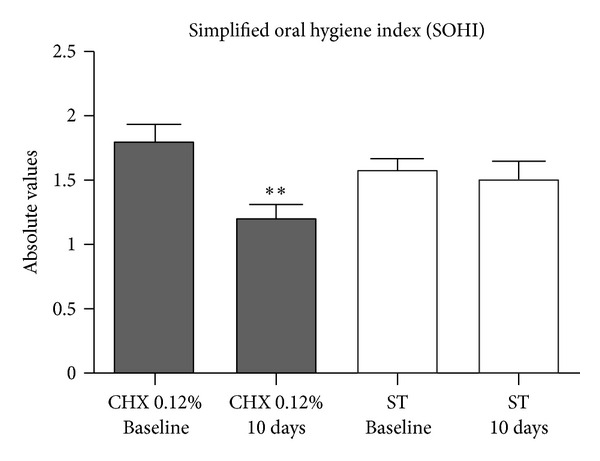
Simplified oral hygiene index (SOHI) values for chlorhexidine 0.12% and *S. terebinthifolius* groups at baseline and after 10-day treatment (**P  value = 0.0036), *Wilcoxon test*.

 Data indicated regular oral hygiene with no significant difference between groups at baseline as regards biofilm accumulation (*P* > 0.05). After 10 days, only the control group showed difference from baseline (*P* < 0.05), as seen in Figure 3 and Table 6.

No adverse effect was reported by the participants or legal guardians during the study period. Under clinical examination, there was no signal of mucosal desquamation and/or tooth staining potentially related to the use of the products.

## 4. Discussion

 Natural products have been used by mankind as a great source of effective therapeutic agents, offering a wide range of biological active molecules [13].

The choice for *S. terebinthifolius* species was based on promising previous *in vitro *data as concerning antibacterial and antiadherent activities against* Streptococcus mutans *and* Lactobacillus casei *[27], antifungal activity on *Candida albicans*, *C. tropicalis,* and *C. krusei * [31, 32], and absence of acute (dose ranging 0.625–5.0 g/kg) and subacute (0.25, 0.625, and 1.5625 g/kg/day) toxic effects of the stem bark on Wistar rats [33]. The international literature does not refer to any mouthwash containing *S. terebinthifolius* in the standards of the present study, which reinforces our pioneering and novelty.

Diseases affecting the periodontium are commonly distributed worldwide [34]. In children, they are extremely important to consider due to consequences for adulthood. In this respect, schoolchildren aged 9–13 years old were enrolled in the present trial. The main reasons for this age range were (i) high gingivitis prevalence [7]; (ii) increased susceptibility to accumulate biofilm, specially erupting elements in infraocclusion; (iii) from 9 years old the child adheres better to treatment protocols, and, more importantly, they have discernment to follow the orientation of not ingesting but only rinsing the product; (iv) schoolchildren usually stay at school for a long time period, often having a cariogenic diet with no oral hygiene.

The use of antimicrobial rinses as adjuncts to mechanical control of dental biofilm and gingival inflammation is well established [9]. Additionally, some clinical trials have demonstrated that the clinical value of a mouthwash to fight gingivitis and accumulation of biofilm in interproximal areas might match or exceed the values found for dental flossing [35, 36]. Therefore, it was chosen to employ *S. terebinthifolius* in a mouthwash formulation, taking into account the ease, speed, and security of administration; substantivity; topic effect desired; attractive visual appearance, aroma, and flavor; low cost.

### 4.1. Treatment Outcomes

The present findings pointed to a potential anti-inflammatory activity of *S. terebinthifolius* mouthwash. There was no significant difference (*P* > 0.05) between the experimental product and 0.12% chlorhexidine after 10 days of 10 mL daily rinses for 01 minute/day.

This species anti-inflammatory property has been previously reported in *in vivo *studies with animals [14, 16]. A recent study demonstrated anti-inflammatory and healing efficacy of *S. terebinthifolius *hydroalcoholic extract (30%) orabase used daily for 14 days to treat Wistar rats' electroproduced wounds [16]. Ribas et al. [14] investigated the therapeutic effects of *S. terebinthifolius* on the tissue healing process of ulcerated oral mucosal wounds in rats. The product was also found to present positive effect on tissue repair.

The biological activity of this species may be related to the presence of phenolic compounds (e.g., catechin tannins) and triterpenoids in the stem bark. The mechanism of action is attributed to selective inhibition of the synthesis of phospholipase A2. Two major components are suggested to be responsible for such property: schinol and masticadienonic acid [37].

Another study assessed the effect of manual periodontal scaling alone and associated with subgingival irrigation on periodontal pockets of patients with chronic periodontitis. The test irrigation products were 0.2% chlorhexidine, a *S. terebinthifolius*-containing solution, and saline. The main clinical parameters evaluated were probing depth, presence of gingival bleeding, and suppuration on probing. Authors concluded that subgingival irrigation with *S. terebinthifolius *once a week for six consecutive weeks may improve clinical effects of the manual scaling [38]. This suggests that this species appears to present activity not only on chronic periodontitis but also on moderate gingivitis as found in the hereby investigation.

Another efficacy variable evaluated was biofilm accumulation. This study found that only 0.12% chlorhexidine was able to reduce significantly the children's amount of biofilm over 10 days. *S. terebinthifolius *was able to mitigate it but did not reach statistical significance. That could be explained probably by the concentration of the experimental product in the formulation. The stem bark tincture presented *in vitro *minimum inhibitory concentration of 0.3125% [27] so that was the concentration employed in the mouthwash. However, due to salivary clearance the product might be diluted to levels not able to exert its proven activity. Further studies are suggested assessing higher concentrations. Moreover, it is reasonable to consider that the SOHI was designed only for index teeth (six in total) and two surfaces (buccal and lingual) [23], while GBI is conducted around all surfaces of all teeth [22]. That might also explain the diverging results between inflammation and biofilm accumulation data in the experimental arm.

Again, there are no clinical studies with this plant species on supragingival biofilm accumulation/removal, but several *in vitro* experiments have pointed to biological potentials.

Alves et al. [31] demonstrated *in vitro* antiadherent activity of *S.terebinthifolius* and 0.12% chlorhexidine upon *Streptococcus mutans*. They found that the plant seems to inhibit glucan synthesis by glycosyltransferase. Accordingly, Freires et al. [27] pointed out that concentrations greater than or equal to 892 *μ*g/mL of the stem bark are able to prevent visible *in vitro* adherence of *Streptococcus mutans* to glass tubes. Further investigations using hydroxyapatite as substrate to evaluate microbial adherence under the action of the product are needed. This type of study is quite important because once bacterial adherence to tooth surfaces or to other microorganisms is not achieved, biofilm cannot be formed properly, which could affect biofilm-induced gingivitis levels.

The antimicrobial activity of the hydroalcoholic extract of *S. terebinthifolius* was evaluated against *S. mutans*, *S. mitis*, *S. sobrinus*, *S. sanguis, *and *Lactobacillus casei*. Bacteriostatic and bactericidal activity was confirmed on these microorganisms [31]. Also, it showed antifungal activity on important fungal species from the oral cavity—*Candida albicans*, *C. tropicalis,* and *C. krusei *[31, 32].

No clinical trial in human beings studying a *S. terebinthifolius* mouthwash to be adjunctive in the treatment of biofilm-induced gingivitis has been reported in the literature. In this respect, our findings give continuity to the studies on this plant species in view of its applicability in the dental practice.

### 4.2. Study Shortcomings and Perspectives

It is pertinent to highlight some limitations of this study in order to subsidize future clinical trials in this field as follows. (i) *Short intervention period.* Enlarging the duration of treatment may be an alternative to assess the effects of prolonged use on oral mucosa and teeth such as desquamation and staining, respectively. In addition, since our findings have indicated a good safety pattern of the product in a 10-day regimen, long-term trials are now encouraged to check: (1) the realignment of the inflammation and the biofilm accumulation indexes; (2) the length of the anti-inflammatory effect of *S. terebinthifolius* mouthwash. (ii) *Superficial sample profiling. *A complete characterization of the sample (e.g., dietary statement, race, and dental experience) would better outline the subjects' profiles as regards clinical and sociodemographic backgrounds. (iii) *Lack of microbiological analysis* such as salivary *S. mutans* counting at baseline and after treatment, considering the *in vitro *antimicrobial potential of the stem bark tincture on this microorganism [27].

Despite that, the present trial is believed to indicate encouraging perspectives on the development of a product containing *S. terebinthifolius* stem bark tincture to be adjunctive in treatment of biofilm-induce gingivitis. Further investigation should consider a more detailed pharmacotechnique analysis of the experimental product and all the aforementioned shortcomings.

 Summarizing, the conditions of the present investigation led to conclude the following. (i) The mouthwash containing *Schinus terebinthifolius* (Brazilian pepper tree) presented significant anti-inflammatory activity after a 10-day use regimen by children having moderate biofilm-induced gingivitis, but it was not able to mitigate biofilm accumulation. (ii) As regards clinical efficacy upon gingivitis, there was no significant difference between the experimental and control groups outcomes after a 10-day treatment. (iii) No adverse effect potentially related to the use of the products was reported or clinically identified.

## 5. Bullet Points

This paper adds the following:

1. a novel alternative for controlling biofilm-induced gingivitis in children/preadolescents other than conventional topical methods;
2. additional informational on the studies about *Schinus terebinthifolius *species, which has been thoroughly investigated worldwide, especially due to its potential for new dental formulations;
3. a specific short-term rinsing protocol to be used and standardized in further clinical investigations with mouthwashes.

This paper is important because of the following.

1. Gingivitis has been largely distributed among children, principally preschoolers. Hence, it becomes important to consider alternatives for better oral health care;
2. A new possibility; to control biofilm-induced gingivitis levels by means of a natural product that seems to overcome adverse effects of synthetic agents such as altered taste and tooth staining is provided.
3. The product investigated was proven to be efficient and safe in a 10-day treatment of moderate gingivitis. Also, it was well accepted by study participants.

## Figures and Tables

**Table 1:** Intra- and interexaminer agreement values obtained by two independent examiners in relation to the study variables.

Variables/indexes	Examiner 1	Examiner 2	Exam. 1 versus exam. 2
Biofilm accumulation	0.816	0.788	0.811
SOHI	Excellent*	Good*	Excellent*

Gingival inflammation	0.795	0.810	0.806
GBI	Good*	Excellent*	Good*

SOHI: simplified oral hygiene index. GBI: gingival bleeding index.

*Agreement stratification according to Landis and Koch [21].

**Table 2:** Pharmaceutical formulations of the experimental and control mouthwashes used in the study.

Formulation	Experimental Arm	Control
Active product	Stem bark tincture of S. terebinthifolius* Concentration: 0.3125%	0.12% chlorhexidine digluconate
Sodium saccharine	0.3%	0.3%
Strawberry smell	0.3%	0.3%
Red dye	0.1%	0.2%
Distilled water	q.s.	q.s.

*Specifications: soluble in water; density: 0.910 g/mL; extractor liquid: hydroalcoholic solution; alcohol strength: 60° GL; dry residue: 2.0%.

**Table 3:** Efficacy variables assessed in the study.

Variable	Outcome of interest	Index employed	Specifications
Gingival inflammation	Primary	Gingival bleeding after probing, Ainamo and Bay [22]	Gentle probing was conducted around all surfaces (buccal, lingual/palatine, mesial, and distal) of all teeth, and values were averaged. Erupting teeth were not considered for assessment. Posteriorly, gingivitis severity was categorized according to the number of bleeding sites (mild: 1–15 sites; moderate: 16–35 sites; severe: equal to or higher than 36 sites)

Biofilm accumulation	Secondary	Simplified oral hygiene index, Greene and Vermillion [23]	Biofilm disclosure was performed (Eviplak, Biodinâmica, Ibiporã, Paraná). Then, values were attributed to index teeth according to the quantity of biofilm found on the buccal or lingual surfaces. Erupting teeth were not considered for assessment, being replaced by the adjacent tooth.

**Table 4:** Clinical profile of the subjects included in the trial according to treatment arm. Values are expressed as percentage, mean, median, and standard deviation (SD).

	Experimental arm *S. terebinthifolius* mouthwash (*n* = 14)	Control arm Chlorhexidine 0.12% (*n* = 13)
Gender		
% Female sex	50.00	69.23
Age (in years)		
Mean ± SD	10.9 ± 0.5	11.2 ± 1.2
Median	11	11
Teeth number		
Mean ± SD	25.6 ± 3.6	24.2 ± 3.3
Median	28	25
Caries risk*		
	Low: 14.29	Low: 7.69
Grouping percentage (%)	Moderate: 7.14	Moderate: 0.00
	High: 0.00	High: 7.69
Caries activity*		
	Low: 71.43	Low: 69.23
Grouping percentage (%)	Moderate: 0.00	Moderate: 0.00
	High: 7.14	High: 15.39
Gingival inflammation activity**		
	Mild: 7.14	Mild: 7.69
Grouping percentage (%)	Moderate: 92.86	Moderate: 92.31
	Severe: 0.00	Severe: 0.00

*According to the clinical-anamnestic method by Krasse [24].

**According to the number of bleeding sites (mild: 1–15 sites; moderate: 16–35 sites; severe: equal to or higher than 36 sites).

**Table 5:** Gingival inflammation levels at baseline and 10 days after using chlorhexidine 0.12% or *S. terebinthifolius* mouthwashes. Values are expressed as means ± standard deviations.

	Experimental arm *S. terebinthifolius *mouthwash (*n* = 14)	Control arm Chlorhexidine 0.12% (*n* = 13)	*P* value
GBI baseline	17.09 ± 5.45^a^	18.59 ± 5.15^a^	0.4710*
GBI after 10 days	11.74 ± 4.03^b^	13.21 ± 6.58^b^	0.4858*
	*P* < 0.0001**	*P* = 0.0027**	

*Paired *t*-test; **unpaired *t*-test.

Different letters in the same column indicate statistically significant differences.

**Table 6:** Biofilm accumulation at baseline and 10 days after using chlorhexidine 0.12% or *S. terebinthifolius* mouthwashes. Values are expressed as means ± standard deviations.

	Experimental arm *S*.* terebinthifolius* mouthwash (*n* = 14)	Control arm Chlorhexidine 0.12% (*n* = 13)	*P* value
SOHI baseline	1.57 ± 0.35^a^	1.80 ± 0.46^a^	0.1998*
SOHI after 10 days	1.53 ± 0.45^a^	1.21 ± 0.34^b^	0.0418*
	*P* = 0.7896**	*P* = 0.0036**	

*Mann-Whitney test; **Wilcoxon test.

Different letters in the same column/row indicate statistically significant differences.
